# Contribution of socio-demographic and clinical characteristics to predict initial referrals to psychosocial interventions in patients with serious mental illness

**DOI:** 10.1017/S2045796024000015

**Published:** 2024-01-29

**Authors:** Guillaume Barbalat, Julien Plasse, Isabelle Chéreau-Boudet, Benjamin Gouache, Emilie Legros-Lafarge, Catherine Massoubre, Nathalie Guillard-Bouhet, Frédéric Haesebaert, Nicolas Franck

**Affiliations:** 1 Centre Ressource de Réhabilitation Psychosociale et de Remédiation Cognitive (CRR), Hôpital Le Vinatier, Centre National de la Recherche Scientifique (CNRS) et Université de Lyon, Lyon, France; 2 Centre Référent Conjoint de Réhabilitation (CRCR), Centre Hospitalier Universitaire de Clermont-Ferrand, Clermont-Ferrand, France; 3 Centre Référent de Réhabilitation Psychosociale et de Remédiation Cognitive (C3R), Centre Hospitalier Alpes Isère, Grenoble, France; 4 Centre Référent de Réhabilitation Psychosociale de Limoges (C2RL), Limoges, France; 5 REHALise, Centre de Réhabilitation Psychosociale, Centre Hospitalier Universitaire de Saint-Etienne, Saint-Etienne, France; 6 Centre de REhabilitation d’Activités Thérapeutiques Intersectoriel de la Vienne (CREATIV), Centre Hospitalier Laborit, Poitiers, France

**Keywords:** clinical features, psychosocial rehabilitation, referrals, socio-demographic features

## Abstract

**Aims:**

Psychosocial rehabilitation (PSR) is at the core of psychiatric recovery. There is a paucity of evidence regarding how the needs and characteristics of patients guide clinical decisions to refer to PSR interventions. Here, we used explainable machine learning methods to determine how socio-demographic and clinical characteristics contribute to initial referrals to PSR interventions in patients with serious mental illness.

**Methods:**

Data were extracted from the French network of rehabilitation centres, REHABase, collected between years 2016 and 2022 and analysed between February and September 2022. Participants presented with serious mental illnesses, including schizophrenia spectrum disorders, bipolar disorders, autism spectrum disorders, depressive disorders, anxiety disorders and personality disorders. Information from 37 socio-demographic and clinical variables was extracted at baseline and used as potential predictors. Several machine learning models were tested to predict initial referrals to four PSR interventions: cognitive behavioural therapy (CBT), cognitive remediation (CR), psychoeducation (PE) and vocational training (VT). Explanatory power of predictors was determined using the artificial intelligence-based SHAP (SHapley Additive exPlanations) method from the best performing algorithm.

**Results:**

Data from a total of 1146 patients were included (mean age, 33.2 years [range, 16–72 years]; 366 [39.2%] women). A random forest algorithm demonstrated the best predictive performance, with a moderate or average predictive accuracy [micro-averaged area under the receiver operating curve from ‘external’ cross-validation: 0.672]. SHAP dependence plots demonstrated insightful associations between socio-demographic and clinical predictors and referrals to PSR programmes. For instance, patients with psychotic disorders were more likely to be referred to PE and CR, while those with non-psychotic disorders were more likely to be referred to CBT and VT. Likewise, patients with social dysfunctions and lack of educational attainment were more likely to be referred to CR and VT, while those with better functioning and education were more likely to be referred to CBT and PE.

**Conclusions:**

A combination of socio-demographic and clinical features was not sufficient to accurately predict initial referrals to four PSR programmes among a French network of rehabilitation centres. Referrals to PSR interventions may also involve service- and clinician-level factors. Considering socio-demographic and clinical predictors revealed disparities in referrals with respect to diagnoses, current clinical and psychological issues, functioning and education.

## Introduction

According to the WHO Rehabilitation Need Estimator, about 190 million people worldwide in 2019 had rehabilitation needs for mental disorders (aggregated as schizophrenia, autism spectrum disorders and developmental intellectual disability) (Institute for Health Metrics and Evaluation, 2022). Psychosocial interventions are at the core of psychiatric recovery. Their goal is to improve social integration, quality of life and overall functioning, by developing emotional, cognitive and social skills (Morin and Franck, 2017). Studies show that psychosocial rehabilitation (PSR) programmes are effective in reducing residual symptoms, increasing everyday life activities (including employment) and reducing the likelihood of psychiatric admissions (Bighelli *et al.*, 2021; Gühne *et al.*, 2015; Huxley and Baldessarini, 2007; McGurk and Mueser, 2004; Morin and Franck, 2017).

A lot of PSR tools are available, such as psychoeducation (PE), family therapy, cognitive behavioural therapy (CBT), peer support, cognitive remediation (CR) or vocational training (VT) (Bighelli *et al.*, 2021; Kern *et al.*, 2009). All these programmes should be considered as evidence-based practices (Huhn *et al.*, 2014; Morin and Franck, 2017), yet there is a paucity of evidence in the literature regarding how clinicians make decisions to refer to PSR interventions based on the needs and characteristics of patients. For instance, meta-analysis and systematic reviews of CR in schizophrenia have reported that, while there is considerable variability in individual treatment response (Biagianti *et al.*, 2021), the identification of response predictors is still an under-investigated topic (Altman *et al.*, 2023). Of note, studies have been criticized for having used small datasets (Corbera *et al*., 2017) and for having reported inconclusive findings (Barlati *et al.*, 2019; Medalia and Richardson, 2005), with no high-quality replicated evidence (Seccomandi *et al.*, 2020). In schizophrenia, a recent meta-analysis concluded that heterogeneous treatment effects of PSR interventions had been explored, but no evident role for any of the moderators (e.g. sex, age, duration of illness and clinical severity) was found (Bighelli *et al.*, 2021).

However, these programmes have different clinical purposes and may not fit patients equally. For instance, the primary objective of PE is to improve insight and therapeutic alliance, while that of CR is to reduce the consequences of cognitive impairments, and that of vocational rehabilitation is to improve social functioning more directly (e.g. via increasing occupational activities). Therefore, it is likely that mental health clinicians try and tailor PSR interventions to the needs and characteristics of patients. For such a highly complex patient population, referrals to PSR interventions would be the product of complex interactions of many different factors, including socio-demographic characteristics, clinical history, current clinical issues and more in-depth psychological factors (e.g. insight, motivation for care and quality of life). Unfolding these interactions would be critical to better understand how these various factors guide clinical decisions. So far, however, studies have used traditional statistical tools that would be unable to take into account such a large number of predictors or their complex relationships with one another.

A potential solution to solve this challenge may be offered by machine learning methods. Machine learning models would typically take into account a vast number of variables and best predict an outcome based on their complex relationships with one another. Moreover, recent advances in explainable artificial intelligence methods have allowed better interpretability of machine learning models with regard to the contribution of each predictive factor (Watson *et al.*, 2019). Here, we used these methods to identify major contributors of initial referrals to four PSR interventions in clinically stable patients with serious mental illness: CBT, CR, PE and VT. Specifically, our aims were to (1) fit a series of machine learning models aiming at predicting referrals to PSR interventions based on socio-demographic and clinical factors; (2) assess the models’ predictive performance and (3) determine the relative importance of the predictors. We reasoned that our approach would help assess whether decisions to refer to PSR interventions depend on socio-demographic and clinical characteristics, evaluate their relative importance when making referrals and highlight disparities in referrals (i.e. identifying populations that are more referred to certain treatment programmes). We used electronic health data from the French network of PSR centres, REHABase, and exploited a relatively large number of socio-demographic and clinical predictors.

## Method

### Data

#### Data source

Our dataset consists of patients enrolled in the REHABase cohort. The cohort includes patients with serious mental illness referred to 15 centres of a French PSR network (Franck *et al.*, 2019). Patients are referred to the centres by public mental health services, private psychiatrists and general practitioners or are self-referred. The vast majority of patients are referred by their secondary care services and continue to benefit from ongoing treatment from their treating team. Referrals are usually accepted based upon clinical assessments, provided that patients demonstrate (1) serious functional impairment resulting from their mental illness, which substantially interferes with one or more major life activities (NIMH, 2023); (2) minimal clinical stability and (3) a clear motivation to attend PSR programmes. Acceptance of referrals is based on clinical judgment and not on clinical or functional scales. Once in the service, patients undergo a standardized socio-demographic, clinical, functional and cognitive evaluation performed by a multidisciplinary team (psychiatrists, nurses, neuropsychologists, occupational therapists and social workers), collected in an electronic case report form. Patients subsequently benefit from a personalized rehabilitation care plan that may last a few months up to a year. Regular group meetings are held monthly to monitor quality control and ensure good inter-rater reliability.

REHABase is a cohort database but, as such, not a study. Therefore, the current study was not pre-planned in the REHABase cohort project. Our analysis was restricted to patients included in the REHABase cohort from January 2016 to January 2022. For the purpose of this study, we only included patients with a DSM-5 (Diagnostic and Statistical Manual of Mental Disorders, 5th edition) diagnosis of schizophrenia spectrum disorder; autism spectrum disorder; bipolar disorder; depressive disorder; anxiety disorder; post-traumatic stress disorder; obsessive compulsive disorder (the latter three disorders were further regrouped under the umbrella of anxiety disorder) or personality disorder, based upon a clinical interview performed by a psychiatrist (American Psychiatric Association, 2013). The database obtained the authorizations required under French legislation (French National Advisory Committee for the Treatment of Information in Health Research, 16.060bis; French National Computing and Freedom Committee, DR-2017-268).

#### Outcome variable

Our outcome variable was clinical referrals to one of the following four PSR programmes:

1. CBT. Under this treatment programme, we regrouped not only standard CBT but also mindfulness and acceptance and commitment therapy, which are often described as the 3rd wave of CBT (Hayes and Hofmann, 2021). CBT aims to help patients overcoming and/or developing awareness of, and accept or let go of, negative thoughts, feelings, physical sensations and behaviours.
2. CR. Methods compiled under this treatment programme aim to improve cognitive abilities via training of cognitive functions such as memory, attention or executive functioning, with the goal of durability and generalization. Four elements are included in CR programmes (Bowie *et al.*, 2020): (1) the presence of an active and trained therapist; (2) the repeated practice of cognitive exercises; (3) the structured development of cognitive strategies and (4) the use of techniques to improve the transfer of cognitive gains to the real world. For the purpose of this study, we included referrals to social cognition programmes in this category. Social cognition programmes focus on emotion processing, social perception, theory of mind skills and/or modifying interpretational cognitive biases.
3. PE. This consists in informing a person with a psychiatric disorder about the bio-psychosocial model of the disorder, its main symptoms, expected effects and side-effects of medication, maintenance treatment, psychotherapy and relapse prevention. This programme is specifically important to increase insight and therapeutic alliance, develop coping strategies and decrease self-stigma.
4. VT. This programme aims at increasing independent functioning and includes recreational activities, volunteering and supported employment (possibly with a period of preparation).

For the purpose of this study, we limited our analysis to the first recorded referrals. Further information is provided in the supplementary methods.

#### Predictors

The choice of predictors was guided by internal discussions among clinical experts of our network and aimed at generating a set of predictors that would best represent socio-demographic and clinical characteristics given the constraints of data availability. Thirty-seven predictors were included. The list of predictors can be found in Table 1. A detailed list of predictors is provided in the supplementary methods). Information on predictors was documented by assessors at baseline (i.e. before the beginning of the treatment).

**Table 1.** Socio-demographic and clinical characteristics of participants

Predictor	CBT (*N* = 318)	CR (*N* = 203)	PE (*N* = 236)	VT (*N* = 389)	Total (*N* = 1146)
**Age, Mean [Min, Max], y**	34.1 [16.6, 58.5]	33.2 [17.0, 59.1]	32.3 [16.3, 60.8]	33.1 [15.5, 71.7]	33.2 [15.5, 71.7]
**Sex**					
Female (%)	141 (44.3%)	82 (40.4%)	77 (32.6%)	149 (38.3%)	449 (39.2%)
Male (%)	177 (55.7%)	121 (59.6%)	159 (67.4%)	240 (61.7%)	697 (60.8%)
**In a relationship**					
Yes (%)	61 (19.2%)	22 (10.8%)	40 (16.9%)	56 (14.4%)	179 (15.6%)
No (%)	248 (78.0%)	178 (87.7%)	194 (82.2%)	330 (84.8%)	950 (82.9%)
Missing (%)	9 (2.8%)	3 (1.5%)	2 (0.8%)	3 (0.8%)	17 (1.5%)
**Education**					
Less than 12 years (%)	96 (30.2%)	100 (49.3%)	75 (31.8%)	163 (41.9%)	434 (37.9%)
12 years or more (%)	212 (66.7%)	101 (49.8%)	158 (66.9%)	220 (56.6%)	691 (60.3%)
Missing (%)	10 (3.1%)	2 (1.0%)	3 (1.3%)	6 (1.5%)	21 (1.8%)
**Unemployment**					
Currently employed (%)	43 (13.5%)	18 (8.9%)	22 (9.3%)	18 (4.6%)	101 (8.8%)
Currently unemployed (%)	273 (85.8%)	185 (91.1%)	212 (89.8%)	368 (94.6%)	1038 (90.6%)
Missing (%)	2 (0.6%)	0 (0%)	2 (0.8%)	3 (0.8%)	7 (0.6%)
**Disabled worker beneficiary**					
No (%)	205 (64.5%)	111 (54.7%)	149 (63.1%)	231 (59.4%)	696 (60.7%)
Yes (%)	93 (29.2%)	79 (38.9%)	67 (28.4%)	137 (35.2%)	376 (32.8%)
Missing (%)	20 (6.3%)	13 (6.4%)	20 (8.5%)	21 (5.4%)	74 (6.5%)
**Social marginalization**					
No (%)	277 (87.1%)	184 (90.6%)	205 (86.9%)	337 (86.6%)	1003 (87.5%)
Yes (%)	30 (9.4%)	11 (5.4%)	22 (9.3%)	43 (11.1%)	106 (9.2%)
Missing (%)	11 (3.5%)	8 (3.9%)	9 (3.8%)	9 (2.3%)	37 (3.2%)
**GAF score, Mean [Min, Max]**	58.5 [21.0, 95.0]	58.4 [20.0, 91.0]	55.9 [10.0, 95.0]	58.9 [9.00, 95.0]	58.1 [9.00, 95.0]
Missing (%)	56 (17.6%)	20 (9.9%)	52 (22.0%)	61 (15.7%)	189 (16.5%)
**Diagnosis**					
ANX (%)	38 (11.9%)	8 (3.9%)	1 (0.4%)	50 (12.9%)	97 (8.5%)
ASD (%)	33 (10.4%)	15 (7.4%)	34 (14.4%)	42 (10.8%)	124 (10.8%)
BAD (%)	45 (14.2%)	22 (10.8%)	32 (13.6%)	38 (9.8%)	137 (12.0%)
DEP (%)	27 (8.5%)	11 (5.4%)	11 (4.7%)	35 (9.0%)	84 (7.3%)
PD (%)	67 (21.1%)	17 (8.4%)	16 (6.8%)	63 (16.2%)	163 (14.2%)
SCZ (%)	108 (34.0%)	130 (64.0%)	142 (60.2%)	161 (41.4%)	541 (47.2%)
**Secondary psychiatric diagnosis**					
No (%)	198 (62.3%)	163 (80.3%)	173 (73.3%)	211 (54.2%)	745 (65.0%)
Yes (%)	120 (37.7%)	38 (18.7%)	63 (26.7%)	178 (45.8%)	399 (34.8%)
Missing (%)	0 (0%)	2 (1.0%)	0 (0%)	0 (0%)	2 (0.2%)
**Addiction diagnosis**					
No (%)	146 (45.9%)	100 (49.3%)	99 (41.9%)	153 (39.3%)	498 (43.5%)
Yes (%)	172 (54.1%)	103 (50.7%)	137 (58.1%)	236 (60.7%)	648 (56.5%)
**Physical health diagnosis**					
No (%)	229 (72.0%)	144 (70.9%)	175 (74.2%)	230 (59.1%)	778 (67.9%)
Yes (%)	84 (26.4%)	54 (26.6%)	53 (22.5%)	153 (39.3%)	344 (30.0%)
Missing (%)	5 (1.6%)	5 (2.5%)	8 (3.4%)	6 (1.5%)	24 (2.1%)
**Number of hospital admission(s)**					
2 or less (%)	165 (51.9%)	97 (47.8%)	113 (47.9%)	222 (57.1%)	597 (52.1%)
3 or more (%)	100 (31.4%)	83 (40.9%)	94 (39.8%)	133 (34.2%)	410 (35.8%)
Missing (%)	53 (16.7%)	23 (11.3%)	29 (12.3%)	34 (8.7%)	139 (12.1%)
**Duration of illness**					
Less than 5 years (%)	96 (30.2%)	54 (26.6%)	86 (36.4%)	134 (34.4%)	370 (32.3%)
5–10 years (%)	52 (16.4%)	47 (23.2%)	49 (20.8%)	74 (19.0%)	222 (19.4%)
More than 10 years (%)	128 (40.3%)	88 (43.3%)	87 (36.9%)	162 (41.6%)	465 (40.6%)
Missing (%)	42 (13.2%)	14 (6.9%)	14 (5.9%)	19 (4.9%)	89 (7.8%)
**Forensic history**					
No (%)	273 (85.8%)	177 (87.2%)	192 (81.4%)	340 (87.4%)	982 (85.7%)
Yes (%)	32 (10.1%)	17 (8.4%)	32 (13.6%)	39 (10.0%)	120 (10.5%)
Missing (%)	13 (4.1%)	9 (4.4%)	12 (5.1%)	10 (2.6%)	44 (3.8%)
**Origin of initial referral**					
Private system (%)	72 (22.6%)	31 (15.3%)	47 (19.9%)	59 (15.2%)	209 (18.2%)
Public system (%)	235 (73.9%)	168 (82.8%)	187 (79.2%)	325 (83.5%)	915 (79.8%)
Missing (%)	11 (3.5%)	4 (2.0%)	2 (0.8%)	5 (1.3%)	22 (1.9%)
**CGI score**					
Mean [Min, Max]	4.14 [1.00, 7.00]	4.12 [1.00, 7.00]	4.26 [1.00, 7.00]	3.79 [1.00, 7.00]	4.04 [1.00, 7.00]
Missing (%)	56 (17.6%)	23 (11.3%)	52 (22.0%)	63 (16.2%)	194 (16.9%)
**SQoL scale (total score)**					
Mean [Min, Max]	45.5 [5.19, 84.4]	51.1 [15.1, 98.4]	51.7 [4.69, 94.3]	50.2 [5.72, 93.8]	49.4 [4.69, 98.4]
Missing (%)	82 (25.8%)	67 (33.0%)	53 (22.5%)	93 (23.9%)	295 (25.7%)
**WEMWBS score**					
Mean [Min, Max]	39.7 [15.0, 65.0]	42.6 [19.0, 69.0]	42.2 [20.0, 67.0]	42.0 [16.0, 70.0]	41.5 [15.0, 70.0]
Missing (%)	62 (19.5%)	37 (18.2%)	37 (15.7%)	61 (15.7%)	197 (17.2%)
**Insight scale (total score)**					
Mean [Min, Max]	8.93 [2.00, 12.0]	8.40 [1.50, 12.0]	8.68 [1.00, 12.0]	8.40 [0, 12.0]	8.60 [0, 12.0]
Missing (%)	123 (38.7%)	64 (31.5%)	59 (25.0%)	149 (38.3%)	395 (34.5%)
**ISMI scale (total score)**					
Mean [Min, Max]	2.34 [1.28, 3.80]	2.24 [1.00, 3.59]	2.19 [1.14, 3.17]	2.26 [1.14, 3.52]	2.26 [1.00, 3.80]
Missing (%)	126 (39.6%)	64 (31.5%)	58 (24.6%)	127 (32.6%)	375 (32.7%)
**Recovery stage**					
Moratorium (%)	46 (14.5%)	24 (11.8%)	27 (11.4%)	54 (13.9%)	151 (13.2%)
Rebuilding (%)	96 (30.2%)	68 (33.5%)	96 (40.7%)	124 (31.9%)	384 (33.5%)
Growth (%)	45 (14.2%)	45 (22.2%)	43 (18.2%)	67 (17.2%)	200 (17.5%)
Missing (%)	131 (41.2%)	66 (32.5%)	70 (29.7%)	144 (37.0%)	411 (35.9%)

Abbreviations: CBT, cognitive behavioural therapy; CR, cognitive remediation; PE, psychoeducation; VT, vocational training; CGI, clinical global impression; SQoL, subjective quality of life; WEMWBS, Warwick-Edinburgh Mental Well-being Scale; ISMI, internalized stigma of mental illness.

#### Missing data

Our dataset was not exempt from missing data (supplementary methods). Missing data were not omitted from the analysis, and participants were not excluded based on missing data. Rather, assuming that information available on our dataset was similarly available to clinicians, we reasoned that missingness may be an important factor when making a decision to refer to a PSR programme. Therefore, for each predictor with missing values, we added an additional ‘missingness indicator’ variable, used as a proxy for unmeasured confounding (Choi *et al.*, 2019). We imputed missing values with the median (for continuous variables) or mode (for categorical variables) of the corresponding variable (Berkelmans *et al.*, 2022; Gmel, 2001; Zhou *et al.*, 2001).

Our final dataset included a total of *N* = 1146 participants (which was similar to the number of initially eligible patients). Finally, note that we did not run any complete cases analysis.

### Analysis

We divided our analysis into two different steps. First, we used various machine learning algorithms to predict referrals to PSR interventions based on socio-demographic and clinical predictors: ridge regression regularization (Friedman *et al.*, 2010), multinomial regression, recursive partitioning trees (Breiman *et al.*, 1984), random forest (Breiman, 2001) and extreme gradient boosting (Chen and Guestrin, 2016). We used a two-step 20-fold cross-validation procedure, also called nested or double cross-validation (Stone, 1974), which resulted in two sets of performance evaluation: (1) an ‘external’ or outer cross-validation performance over 20 testing folds and (2) a range of 20 ‘internal’ or inner cross-validation performance. Predictive accuracy was assessed based on the micro-averaged area under the receiver operating curve (AUROC) and the area under the precision-recall curve (AUPRC). Details related to data preprocessing, grid search for machine learning hyperparameters, cross-validation strategy and measures of predictive accuracy are reported in the supplement (supplementary methods, Supplementary Table S2).

Second, we used explainable machine learning methods from the best performing algorithm to compute the explanatory power of each socio-demographic and clinical feature. To do so, we used the artificial intelligence-based SHAP (SHapley Additive exPlanations) method, regarded as the only model-agnostic explanation method with a solid theoretical foundation (Lundberg and Lee, 2017) (supplementary methods). For each variable and each observation, a higher SHAP value corresponds to a higher likelihood of the target outcome (i.e. being referred to one of the four PSR treatment programmes). We first provided predictor-level SHAP absolute values aggregated at the level of the population in order to rank the predictors’ overall predictive ability (aka variable importance). Second, for each treatment programme, we provided one-way dependence plots showing the association between the raw values of the 10 most important predictors and their SHAP values.

To perform this analysis, we used R version 4.1 and packages such as caret, multiROC and fastshap. The Prediction model Risk Of Bias ASsessment Tool (PROBAST) guided writing of the manuscript (Wolff *et al.*, 2019).

## Results

The mean age of the overall cohort was 33 years (range, 16–72 years), and 449 (39%) were women. Of those, 318 (27.8%) were referred to CBT, 203 (17.7%) to CR, 236 (20.6%) to PE and 388 (33.9%) to VT. A complete description of the dataset is provided in Table 1.

### Performance of machine learning models

The highest overall micro-cross-validated AUROC and AUPRC were obtained for the random forest model (AUROC: external validation, 0.672; internal validation across 20 folds: range, 0.659–0.674; PR-AUC: external validation, 0.407; internal validation across 20 folds: range, 0.381–0.410; Supplementary Figure S1). Random forest outperformed regression regularization (AUROC, external validation: 0.668); multinomial regression (0.662); extreme gradient boosting (0.653) and recursive partitioning trees (0.638) (Supplementary Tables S3 and S4).

### Contribution of each predictor to initial referrals

We used the artificial intelligence-based SHAP method to further explore the explanatory power of socio-demographic and clinical variables. Aggregating the four programmes, the 10 most important contributors to the prediction on a global scale were as follows: having a diagnosis of schizophrenia; having a secondary psychiatric diagnosis; education; having a physical health diagnosis; unemployment; being a disabled worked beneficiary; having a diagnosis of personality disorder; score on the clinical global impression (CGI) scale; missing information on insight and sex (Supplementary Figure S2).

**Figure 1.**
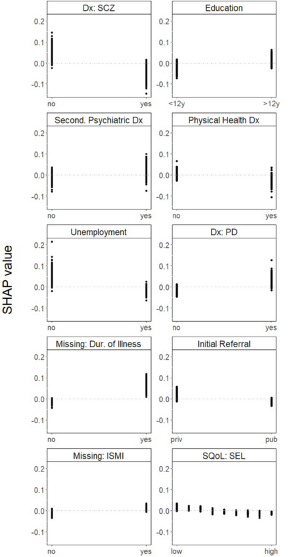
One-way SHAP dependence plot of the 10 most important predictors of referrals to CBT.

Abbreviations: CBT, cognitive behavioural therapy; SHAP, SHapley Additive exPlanations; Dx, diagnosis; SCZ, schizophrenia spectrum disorders; Second., secondary; PD, personality disorders; Dur., duration; ISMI, internalized stigma of mental illness; SQoL, subjective quality of life; SEL, self-esteem. Values of the predictor are represented on the x-axis. SHAP values are represented on the y-axis. A higher SHAP value indicates a higher likelihood of referral to CBT.

We then examined associations between the predictors and initial referrals to PSR interventions using one-way SHAP dependence plots. A higher likelihood of being referred to CBT was associated with not having a diagnosis of schizophrenia; having a relatively high level of education; having a secondary psychiatric diagnosis; not having a physical health diagnosis; not being unemployed; having a diagnosis of personality disorder; not having been referred by a clinician from the public system and having a relatively low self-esteem. Missing information on duration of illness and missing information on self-stigma were also associated with being referred to CBT (Fig. 1).

**Figure 2.**
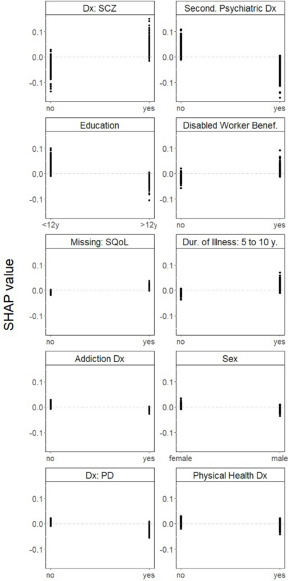
One-way SHAP dependence plot of the 10 most important predictors of referrals to CR.

Abbreviations: CR, cognitive remediation (which for the purpose of this study was grouped with social cognition); SHAP, SHapley Additive exPlanations; Dx, diagnosis; SCZ, schizophrenia spectrum disorders; Second., secondary; Benef., beneficiary; SQoL, subjective quality of life; Dur., duration; PD, personality disorders. Values of the predictor are represented on the x-axis. SHAP values are represented on the y-axis. A higher SHAP value indicates a higher likelihood of referral to CR.

A higher likelihood of being referred to CR was associated with having a diagnosis of schizophrenia; not having a secondary psychiatric diagnosis; having a relatively low level of education; being a disabled worker beneficiary; having an illness duration of 5–10 years; not having an addiction diagnosis; being female; not having a diagnosis of personality disorder and not having a physical health diagnosis. Missing information on quality of life was also associated with being referred to CR (Fig. 2).

**Figure 3.**
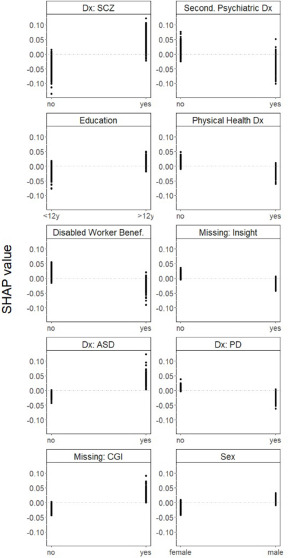
One-way SHAP dependence plot of the 10 most important predictors of referrals to PE.

Abbreviations: PE, psychoeducation; SHAP, SHapley Additive exPlanations; Dx, diagnosis; SCZ, schizophrenia spectrum disorders; Second., secondary; Benef., beneficiary; ASD, autism spectrum disorders; PD, personality disorders; CGI, clinical global impression. Values of the predictor are represented on the x-axis. SHAP values are represented on the y-axis. A higher SHAP value indicates a higher likelihood of referral to PE.

A higher likelihood of being referred to PE was associated with having a primary diagnosis of schizophrenia; not having a secondary psychiatric diagnosis; having a relatively high level of education; not having a physical health diagnosis; not being a disabled worker beneficiary; having a primary diagnosis of autism spectrum disorder; not having a primary diagnosis of personality disorder and being male. Missing information on insight was associated with *not* being referred to PE, while missing information on CGI was associated with being referred to PE (Fig. 3).

**Figure 4.**
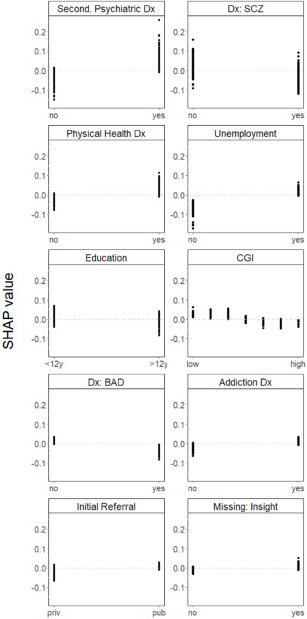
One-way SHAP dependence plot of the 10 most important predictors of referrals to VT.

Abbreviations: VT, vocational training; SHAP, SHapley Additive exPlanations; Second., secondary; Dx, diagnosis; SCZ, schizophrenia spectrum disorders; CGI, clinical global impression; BAD, bipolar affective disorders. Values of the predictor are represented on the x-axis. SHAP values are represented on the y-axis. A higher SHAP value indicates a higher likelihood of referral to VT.

Finally, a higher likelihood of being referred to VT was associated with having a secondary psychiatric diagnosis; not having a primary diagnosis of schizophrenia; having a physical health diagnosis; being unemployed; having a relatively low level of education; having a low score on the CGI scale; not having a primary diagnosis of bipolar disorder; having an addiction disorder and having been initially referred by a clinician from the public system. Missing information on insight was also associated with being referred to VT (Fig. 4).

## Discussion

The current study used a large number of socio-demographic and clinical variables to predict referrals to four PSR treatment programmes in patients with serious mental illness among a network of 15 rehabilitation centres. We first show that the predictive accuracy of a random forest algorithm outperformed that of recursive partitioning trees, extended gradient boosting, and to a lesser extent, multinomial regression and regularized regression. Overall, the performance of our winning random forest algorithm (AUROC of 0.67) may be considered as either moderate, fair or acceptable or even poor, weak or low (de Hond *et al.*, 2022). It is unlikely that such uncertainty indicates a poor model or lack of access to important predictors, given that we used several machine learning algorithms and that we had access to an overall large number of predictors. A more reliable hypothesis may be that available socio-demographic and clinical features are necessary but not sufficient to explain initial decisions to refer patients to PSR programmes. Referrals might also rely on other important factors in the dynamics of clinical decisions, for instance, centre characteristics (such as being a teaching hospital, having its own culture regarding treatment referral, financial resources, etc.), clinicians heuristics, provider bias (e.g. implicit or explicit bias) and patients’ preferences (Blankertz and Robinson, 1996; Carter *et al.*, 2015; Medalia and Richardson, 2005). Another possibility is that lack of resources for certain treatment programmes may lead to patients being referred to interventions that would not have been chosen as a first instance. An interesting goal for future research would be to tease out the contribution of the above-mentioned factors to predict referrals to PSR interventions.

The moderate contribution of socio-demographic and clinical characteristics to predict referrals to PSR interventions is enlightened by the implementation science literature, which studies ‘methods to promote the systematic uptake of […] evidence-based practice into routine practice’ (Eccles and Mittman, 2006). Within this framework, some of the non-patient variables that we mentioned above may be understood as barriers to implementing psychosocial interventions. Psychosocial and recovery interventions are indeed relatively difficult to implement, due to being too complex and time-consuming or requiring significant material, financial and human resources (van der Krieke *et al.*, 2015). Interestingly, blockers of PSR interventions may themselves be moderated by patients’ socio-demographic and clinical characteristics. For instance, compared to those with depressive or anxiety disorders, patients with schizophrenia may not be referred to CBT because of the beliefs that therapy may be difficult and cumbersome, especially when considering lack of resources or training, and the fact that symptoms may impede engagement (Hazell *et al.*, 2017; Ince *et al.*, 2016; Prytys *et al.*, 2011).

Despite its moderate performance, our winning random forest model likely provided the *best possible* predictive accuracy with respect to the data at hand. This was further exploited using artificial intelligence-based SHAP values (Lundberg and Lee, 2017) to decipher the contribution of socio-demographic and clinical factors to predict initial referrals to PSR interventions. Given the paucity of evidence regarding how patients’ needs and characteristics guide referrals, any major disparities would reflect informal, ‘eminence-based’ or circumstantial practices that would rely mostly on our clinicians’ expert opinions. Incidentally, these choices may also reflect unmet patient needs, where some categories of patients may benefit from suboptimal treatment programmes. We wish to encourage clinicians and researchers to further investigate these circumstantial practices (described below), for instance, by determining whether they are ethical and optimal from a health services perspective. Finally, because our findings may reflect unmet needs rather than good practice per se, we can only, at this point, recommend to further explore these aspects, rather than directly use our findings in clinical practice.

A first disparity may be related to psychiatric diagnosis and comorbidities. While all treatment programmes tested in the current study have been recommended in patients with schizophrenia (Crowther *et al.*, 2001; Morin and Franck, 2017; Twamley *et al.*, 2003; Watzke *et al.*, 2009), PE and CR were more likely to be chosen for patients with psychotic disorders than CBT and VT. Patients with psychosis may be seen as having higher needs for (and higher gains from) PE (de Barros Pellegrinelli *et al.*, 2013) and CR (Li *et al.*, 2020), due to their well-documented lack of insight (Braw *et al.*, 2012; Lysaker *et al.*, 2018; Ramachandran *et al.*, 2016) and cognitive difficulties (Zaytseva *et al.*, 2018), respectively. Clinicians also preferred to refer patients without comorbidities to the former programmes, perhaps in the view of facilitating treatment delivery (Gold *et al.*, 2020; Thornicroft *et al.*, 2019). This was in sharp contrast with referrals to VT, where comorbidities, whether from addictions, mental or physical health, would not *a priori* play a role in treatment completion.

We also report disparities relative to clinical characteristics. For instance, the fact that patients referred to VT were more likely to have a lower severity score on the CGI scale may reflect the need to satisfy the demands of occupational activities or supported work. By contrast, current clinical and psychological issues (in particular low self-esteem) would increase the likelihood of referrals to CBT (Carter *et al.*, 2015; Keeley *et al.*, 2008; Maddox *et al.*, 2019), which primarily aims to address current negative thoughts about self and others.

Functioning and educational attainment were other sources of disparities. For instance, clinicians tend to refrain from referring patients with social dysfunctions (e.g. unemployment and being a disabled worker beneficiary) and lack of education to CBT and PE, as they may judge that these treatment programmes may require a certain level of literacy and understanding (Hu *et al.*, 2022). By contrast, unemployed patients were preferentially referred to VT (which provides direct occupational support), and patients who are disabled worker beneficiaries were more likely to be referred to CR (which would tackle cognitive dysfunction in relation to the disability). Likewise, the fact that a relatively low level of education was associated with referrals to CR may be a reflection that these treatment programmes aim at palliating for cognitive and social deficits (Akshaya *et al.*, 2022) that are known to be strongly associated with lack of educational attainment (Dalsgaard *et al.*, 2020; Guerra-Carrillo *et al.*, 2017; Lövdén *et al.*, 2020).

### Limitations

First, our results are subject to some degree of uncertainty due to the substantial amount of missing values, especially for clinical features (e.g. clinical severity, insight, quality of life, well-being and self-stigma). Missing information, however, was a significant contributor to initial referrals, where, for instance, PE was not the treatment of choice when insight – the key process addressed by this intervention – had not been evaluated.

Second, one might question the generalizability of our findings. Indeed, delivering some of the interventions analysed in the present study in low-resource countries may be unfeasible. Likewise, some of our predictors were directly linked to the French universal healthcare system and social security (e.g. being a disabled worker beneficiary), and future studies should test whether these criteria are meaningful to predict referrals to PSR programmes in other countries. Finally, our analysis may only be applicable to patients that consent to have their data recorded in our database. As we do not record patients who refuse to participate, we have no possible way of investigating whether this may have been a potential problem for our analysis. Informal discussions with mental health clinicians, however, indicate that patients who do not consent to have their data collected are rare.

Third, variable importance measures should not be interpreted as *absolute* indicators of the contribution of individual variables. Machine learning algorithms, such as random forest, involve non-linear transformations and interactions between variables; therefore, each variable importance measure is in essence *relative* to other variables.

Fourth, in theory, an analysis taking into account multiple referrals rather than initial referrals only would have been both more representative of referrals as they happen in real life and more statistically informative (i.e. with greater statistical power). Further, such an analysis would enable the investigation of socio-demographic and clinical factors that contribute to greater resource utilization. Such an analysis, however, would have been both more complex (taking into account censored data) and uncertain (due to the amount of missing observations for clinical variables after the initial referral).

Fifth, this study focused on treatment referral rather than treatment effectiveness. Choices made by experts from our network may not necessarily be associated with positive outcomes. As mentioned above, it is not impossible that they reflect, at least in part, service- or clinician-level factors, rather than evidence-based patient-level criteria. In that sense, future studies should investigate whether features that contribute to treatment referrals are also predictive of positive treatment outcomes.

## Conclusion

Using a variety of machine learning models, we demonstrated that a combination of socio-demographic and clinical features was not sufficient to accurately predict initial referrals to four PSR programmes among a French network of rehabilitation centres. In addition, the explanations generated by SHAP plots provided valuable insights into the sources of referrals for our cohort of patients and, in particular, disparities in referrals with respect to diagnoses, current clinical and psychological issues, functioning and education. This, in turn, may provide potential avenues for future research aiming at investigating and resolving such disparities.

## Supporting information

Barbalat et al. supplementary material Barbalat et al. supplementary material

## Data Availability

The datasets generated during and/or analysed during the current study are available upon reasonable request to the corresponding author, GB.

## Supplementary material

The supplementary material for this article can be found at https://doi.org/10.1017/S2045796024000015.
