# Supplementary material for: Contribution of socio-demographic and clinical characteristics to predict initial referrals to psychosocial interventions in patients with serious mental illness
Source: Epidemiol Psychiatr Sci. 2024 Jan 29;33:e2. doi: 10.1017/S2045796024000015 (PMC10894705; doi:10.1017/S2045796024000015)
Supplement: Barbalat et al. supplementary material [file S2045796024000015sup001.docx]

**Supplemental Materials**

G Barbalat, J Plasse, I Chéreau-Boudet, et al. Supplementary Materials for Contribution of socio demographic and clinical features to predict initial referrals to psychosocial interventions in patients with serious mental illness.

**Supplementary Methods.**

**Supplementary Table 1. Main characteristics of the four psychosocial interventions**

**Supplementary Table 2. Hyperparameter grid for each machine learning algorithm**

**Supplementary Table 3. AUROCs of the external Cross-Validation Set for each machine learning algorithm**

**Supplementary Table 4. AUPRCs of the external Cross-Validation Set for each machine learning algorithm**

**Supplementary Figure 1. AUROCs and AUPRCs of the external Cross-Validation Set for the winning random forest algorithm**

**Supplementary Figure 2. Variable Importance Plot for all predictors of referrals to CBT, CR, PE and VT**

**Supplementary References.**

# **Supplementary Methods**

Outcome variable (psychosocial interventions)

Interventions were explicitly coded as such on our electronic database. Programs are typically run by trained psychologists (neuropsychologists for CR) and/or nurses, with occasional participation of peer support workers (for PE), and occupational therapists or social workers (for VT). All four interventions were done in individual or group settings, and lasted about 4 months on average (**Supplementary Table 1**). 54.8% of patients had to wait 3 months or less, and 72.1% of patients had to wait 6 months or less, before the start of their treatment program. We excluded other programs, such as social skills training, health education, family therapy, support or education, and peer support because of lack of observations.

On average, referrals to more than one program occur in 77.5% of patients. Yet, note that for the purpose of this study, we limited our analysis to the first recorded referrals: if patients were referred to a sequence of consecutive interventions, only the first referral was considered (one observation per participant). This decision was rationalized by the fact that we anticipated that follow-up referrals would be subject to clinician-related factors such as therapeutic drift and infidelity to treatment manuals, rather than socio-demographic and clinical characteristics per se. Our choice was also a pragmatic decision as an analysis taking into account multiple (following) referrals would have been subject to several missing observations. Finally, note that patients were not referred to multiple programs at the same time, or very closely in time.

Predictors

- Socio-demographic information: age; sex (male vs. female); being in a relationship (yes vs. no); education (less than 12 years vs. 12 years or more). Other socio-demographic features included functioning: unemployment (yes vs. no); being a disabled worker beneficiary (yes vs. no); social marginalization (based on clinician judgment of a patient’s level of desocialization at the time of assessment, i.e. withdrawal from social contacts at the time of assessment: yes vs. no); score on the Global Assessment of Functioning (GAF) scale (American Psychiatric Association, 2013).

Race/ethnicity and gender identity (how individuals perceive themselves) and sexual orientation (their emotional, romantic or sexual attraction to other people) were not included in our set of predictors as it is not allowed to systematically collect such sensible data in France.

- Clinical information: primary psychiatric diagnosis (anxiety disorder (reference); autism spectrum disorder; bipolar disorder; depressive disorder; personality disorder; schizophrenia spectrum disorder); having a secondary (comorbid) psychiatric diagnosis (yes vs. no); having an addiction diagnosis (DSM-5 substance use disorders and/or behavioral addiction: yes vs. no); having a physical health diagnosis (yes vs. no); number of psychiatric admissions (2 or less vs. 3 or more); duration of illness (less than 5 years vs. 5 to 10 years vs. more than 10 years); forensic history (yes vs. no); origin of initial referral (referrer from the private vs. public system); severity of illness score on the Clinical Global Impression (CGI) scale (Guy 1976). We also included information from self-reported scales and questionnaires on: quality of life (Subjective Quality of Life scale 18 (SQoL18) - total score and 8 subscores: self-esteem, autonomy, resilience, relationships with friends, relationships with family, romantic relationships, physical and mental well-being; (Boyer et al., 2010); well-being total score (Warwick-Edinburgh Mental Well-Being Scale (WEMWBS); (Tennant et al., 2007); insight (Birchwood insight scale: total score and 3 subscores – awareness of illness, need for treatment, re-labeling of symptoms; (Birchwood et al., 1994); self-stigma (Internalized Stigma for Mental Illness – total score and 5 subscores: alienation, stereotype endorsement, discrimination experience, social withdrawal, stigma resistance; (Boyd Ritsher et al., 2003); and recovery stage (STages of Recovery Instrument (STORI); (Andresen et al., 2006) – scores were transformed into 3 categories: moratorium (reference), rebuilding and growth).

Note that center dummies were not included in our set of predictors. Indeed, the current study aimed to investigate socio-demographic and clinical contributors to treatment referrals, irrespective of the characteristics of treatment centers.

Missing data

Missing values either resulted from non-recording, or if recording occurred after the PSR intervention was commenced. Missing data were less frequent for socio-demographic variables (with a maximum of 1.8% of missing values for education), than clinical and psychological variables (with a maximum of 35.9% of missing values for recovery stage). We hypothesized missingness not at random (MNAR), where missing values may not be predicted based on the other collected variables (e.g. would result from unmeasured confounding) (Rubin 1976). We reasoned that such missingness would be due to an outside factor not reflected in the dataset, for instance: who is collecting the data, participants not willing to respond, or the nature or value of the missing variable itself (e.g. because collecting these data is more cumbersome, or would involve sensitive information). Another, non-mutually exclusive possibility is that missingness is associated with the socio-demographic and clinical variables included in the dataset.

Standard machine learning analysis

We ran a series of machine learning algorithms aiming to optimally predict referrals to one of the four treatment programs, using the predictors described above.

Preprocessing involved removing correlated predictors (ρ > 0.9), as well as predictors with near-zero values. Over an initial number of 80 predictors, 27 predictors were removed, with 53 predictors remaining for model development. Continuous predictors were not dichotomized.

We used the following algorithms: ridge regression regularization (Friedman et al., 2010), multinomial regression, recursive partitioning trees (Breiman et al., 1984), random forest (Breiman 2001), extreme gradient boosting (Chen & Guestrin, 2016). To minimize computational time for the more complex algorithms that include several hyperparameters (random forest, gradient boosting), we used a resampling “adaptive” scheme that was found effective at finding reasonable values of tuning parameters in a more computationally effective way (Kuhn 2014). The grid search of hyperparameters for each algorithm is reported in **Supplementary Table 2.**

To minimize over-fitting, we chose to split the dataset into K=20 independent folds (Phillips et al., 2022), using repeatedly (20 times) 19 folds for model training and one fold for model testing, and ensuring that testing occurred on unseen data. For each treatment program, each of the 20 folds comprised 56-58 observations, meaning that, for each of the 20 iterations, 1088-1090 observations were in the training set, and 56-58 observations were used for testing the model. We ensured that each fold included the same proportion of patients referred to each of the four treatment programs (ranges for the training folds: CBT, 27.7-27.8%; CR, 17.6-17.7%; PE, 20.6-20.7%; VT, 33.8-34.0%; ranges for the testing folds: CBT, 26.3-28.6%; CR, 17.2-19.3%; PE, 19.0-21.1%; VT, 32.8-35.1%). Finally, we ensured that each fold included similar observations from one machine learning algorithm to the other.

The 19 folds used for model training were also subject to a 20 folds cross-validation procedure, with 10 repeats to minimize instability. Overall, this 2-steps cross-validation procedure, also called nested or double cross-validation (Stone 1974), resulted in two sets of performance evaluation: (1) an “external” or outer cross-validation performance over the 20 testing folds (i.e. over the N=1146 observations); and (2) a range of 20 “internal” or inner cross-validation performance over 19 training folds (i.e. 20 times over N=1088-1090 observations).

For each algorithm, training consisted in selecting the best set of hyperparameters (aka hyperparameter tuning), which was further used for testing the model. Hyperparameter tuning aimed to optimize the area under the receiving operating curve (AUROC). We also reported the area under the precision-recall curve (AUPRC), The difference between the AUROC and the AUPRC is that, as the threshold probability by which the model’s predictions are actually classified into PSR interventions varies from 0 to 1, the curve is defined by a trade-off between sensitivity and 1-specificity for the AUROC, vs. between precision (i.e. positive predictive value) and recall (i.e. sensitivity) for the AUPRC. The AUROC is perhaps more popular but the AUPRC would better take into account imbalance datasets since precision depends on the prevalence of the observed outcomes. Likewise, we report both the macro- and micro-average of the AUROC and AUPRC. Briefly, a macro-average computes the metric independently for each class and then calculates the raw average, treating all classes equally. A micro-average takes into account the prevalence of all classes to calculate the metric, i.e. a micro-average would better take into account class imbalance than a macro-average.

Explainable machine learning analysis

Using the best performing algorithm, we aimed to determine the explanatory power of each predictor with respect to referrals to PSR treatment programs. To do so, we used an artificial intelligence SHAP (SHapley Additive exPlanations) method (Lundberg & Lee, 2017). In short, SHAP values calculate the importance of a feature by comparing what a model predicts with and without the feature. Of existing work on interpreting individual predictions, SHAP is regarded as the only model-agnostic explanation method with a solid theoretical foundation (Lundberg & Lee, 2017).

A SHAP value is the average marginal contribution of a feature value across all possible sets of features, which is computationally very costly. The *fastshap* R package provides an efficient way to reduce the computational cost of computing SHAP values, by calculating approximated SHAP values using a Montecarlo simulation approach (Štrumbelj & Kononenko, 2014; Lundberg & Lee, 2017; Greenwell, 2021).

**Supplementary Table 1. Main characteristics of the four psychosocial interventions**

|  | **CBT** | **CR** | **PE** | **VT** | **Overall** |
| --- | --- | --- | --- | --- | --- |
|  | (N=318) | (N=203) | (N=236) | (N=389) | (N=1146) |
|  |  |  |  |  |  |
| **Intervention done in group setting (%)** | 211 (66.4%) | 89 (43.8%) | 165 (69.9%) | 205 (52.7%) | 670 (58.5%) |
| Missing (%) | 11 (3.5%) | 18 (8.9%) | 27 (11.4%) | 31 (8.0%) | 87 (7.6%) |
|  |  |  |  |  |  |
| **Median duration of intervention (hr)** | 16 | 20 | 14 | Estimated range:  8-24 | 15 |

Abbreviations: CBT, cognitive and behavioural therapy; CR, cognitive remediation; PE, psychoeducation; VT, vocational training.

**Supplementary Table 2. Hyperparameter grid for each machine learning algorithm**

| **Machine Learning algorithm** | **method argument in *caret* R package** | **Hyperparameters range** |
| --- | --- | --- |
| Regression regularization | glmnet | alpha=0  lambda range= [0.0077-77.96] (100 values) |
| Multinomial regression | multinom | decay=seq(from=0, to=1, by=0.1) |
| Recursive partitioning trees | rpart | cp=seq(from=0, to=.02, by=.0001) |
| Random forest | ranger | mtry range=[1-53]  splitrule: {“extratrees”; “gini”}  min.node.size range=[1-20]  tuneLength=100 |
| Extended gradient boosting | xgboost | eta range=[0.0071-0.5882]  max_depth range=[1-10]  gamma range=[0.0571-9.931]  colsample_bytree range=[0.3031-0.6983]  min_child_weight range=[0-20]  subsample range=[0.2516-0.9994]  tuneLength=100 |

Pre-defined using *glmnet* R package

Pre-defined based on discussions with machine learning experts

Obtained from resampling “adaptive” scheme

**Supplementary Table 3. AUROCs of the external Cross-Validation Set for each machine learning algorithm**

| **Algorithm** | **CBT** | **CR** | **PE** | **VT** | **Macro-av.** | **Micro-av.** |
| --- | --- | --- | --- | --- | --- | --- |
| Regression regularization | 0.626 | 0.637 | 0.665 | 0.649 | 0.644 | 0.668 |
| Multinomial regression | 0.609 | 0.638 | 0.662 | 0.648 | 0.639 | 0.662 |
| Recursive partitioning trees | 0.605 | 0.585 | 0.584 | 0.587 | 0.59 | 0.638 |
| Random forest | 0.651 | 0.639 | 0.661 | 0.641 | 0.648 | 0.672 |
| Extended gradient boosting | 0.616 | 0.616 | 0.66 | 0.631 | 0.631 | 0.653 |

Abbreviations: AUROC, area under the receiver operating characteristics; CBT, cognitive and behavioral therapy; CR, cognitive remediation; PE, psychoeducation; VT, vocational training; Macro-av., macro-average; Micro-av, micro-average.

Range (median) of (best) hyperparameters for the winning random forest model across the 20 folds: mtry: 4-27 (14); splitrule: extratrees; min.node.size: 16-20 (18).

**Supplementary Table 4. AUPRCs of the external Cross-Validation Set for each machine learning algorithm**

| **Algorithm** | **CBT** | **CR** | **PE** | **VT** | **Macro-av.** | **Micro-av.** |
| --- | --- | --- | --- | --- | --- | --- |
| Regression regularization | 0.378 | 0.46 | 0.288 | 0.312 | 0.359 | 0.388 |
| Multinomial regression | 0.372 | 0.456 | 0.274 | 0.306 | 0.352 | 0.378 |
| Recursive partitioning trees | 0.605 | 0.585 | 0.584 | 0.587 | 0.59 | 0.638 |
| Random forest | 0.412 | 0.483 | 0.288 | 0.316 | 0.375 | 0.407 |
| Extended gradient boosting | 0.365 | 0.456 | 0.268 | 0.291 | 0.345 | 0.376 |

Abbreviations: AUPRC, area under the precision recall curve; CBT, cognitive and behavioral therapy; CR, cognitive remediation; PE, psychoeducation; VT, vocational training; Macro-av., macro-average; Micro-av, micro-average.

Range (median) of (best) hyperparameters for the winning random forest model across the 20 folds: mtry: 4-27 (14); splitrule: extratrees; min.node.size: 16-20 (18).

**Supplementary Figure 1. AUROCs and AUPRCs of the external Cross-Validation Set for the winning random forest algorithm.**


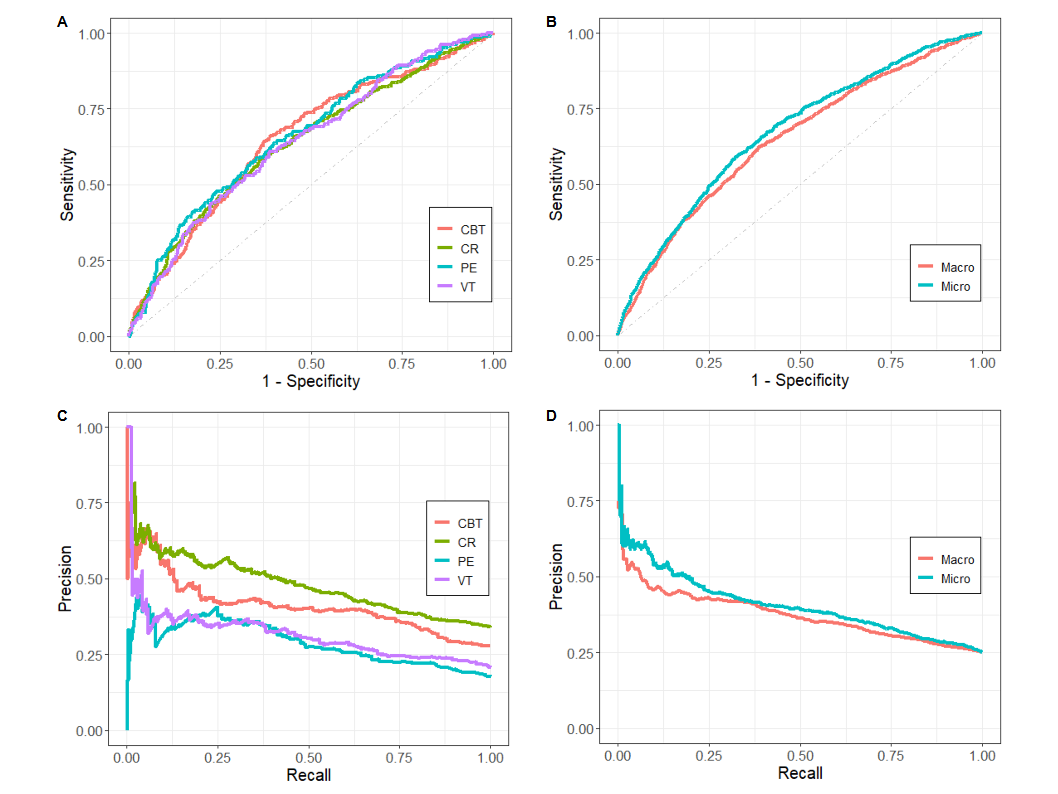
Abbreviations: AUROC, area under the receiver operating characteristics; AUPRC, area under the precision recall curve.

**Supplementary Figure 2. Variable Importance Plot for all predictors of referrals to CBT, CR, PE and VT**


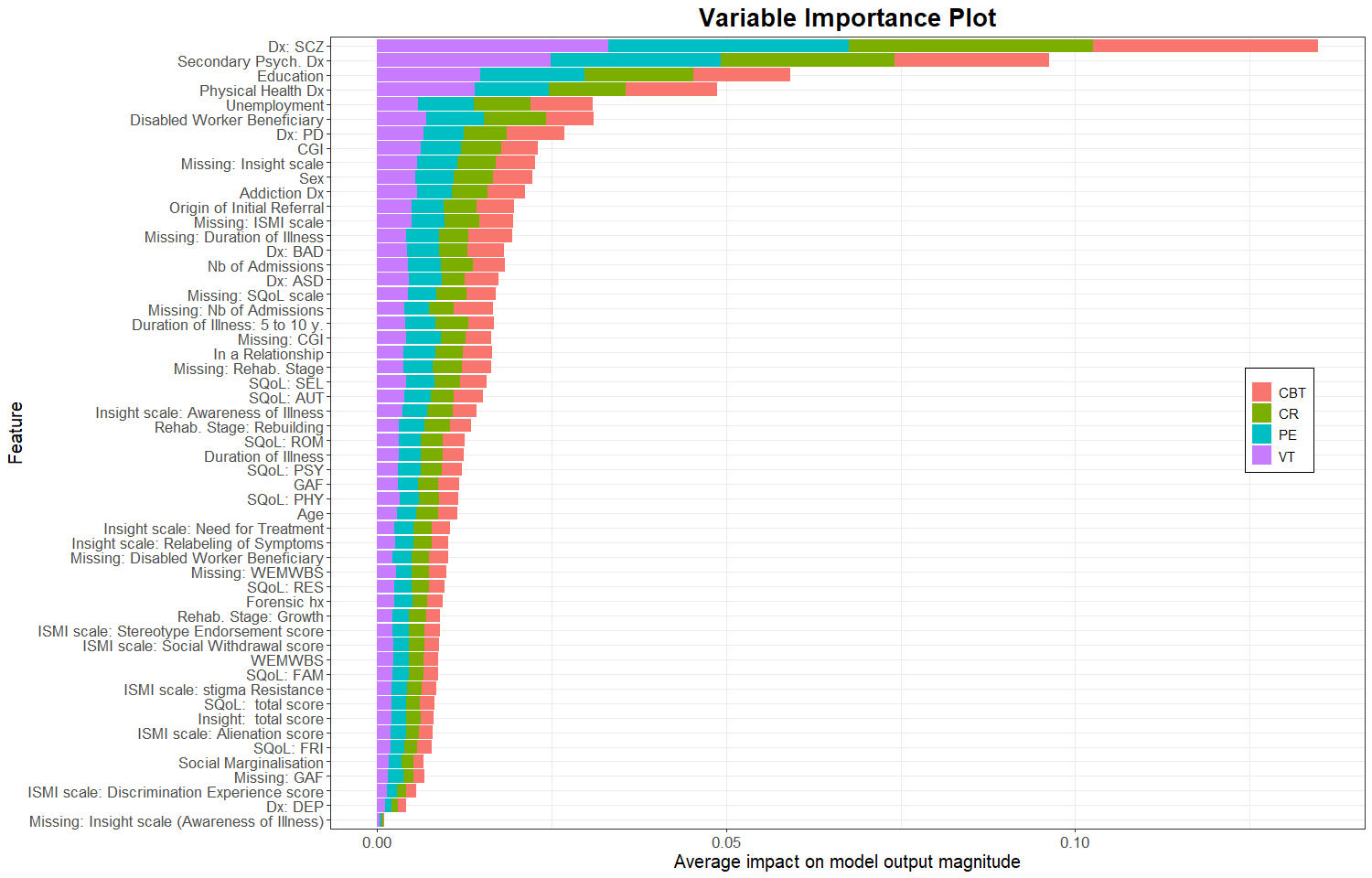


Abbreviations: CBT, cognitive and behavioral therapy; CR, cognitive remediation; PE, psychoeducation; VT, vocational training; GAF, global assessment of functioning; ASD, autism spectrum disorders; BAD, bipolar affective disorders; DEP, depressive disorders; PD, personality disorders; SCZ, schizophrenia spectrum disorders; CGI, clinical global impression; SQoL, subjective quality of life; WEMWBS, Warwick-Edinburgh mental well-being scale; ISMI, internalized stigma of mental illness; Dx, diagnosis; Psych., psychiatric; Nb, number; Rehab, rehabilitation; hx, history; AUT, autonomy; RES, resilience; FRI, relationships with friends; FAM, relationships with family; ROM, romantic relationships; SEL, self-esteem; PHY, physical well-being; PSY, psychological well-being.

Averaged absolute SHAP values are represented on the x-axis. Predictors are represented on the x-axis.

**Supplementary References**

**Andresen R, Caputi P and Oades L** (2006) Stages of recovery instrument: development of a measure of recovery from serious mental illness. *Australian & New Zealand Journal of Psychiatry* **40**, 972–980

**Birchwood M, Smith J, Drury V, Healy J, Macmillan F and Slade M** (1994) A self-report Insight Scale for psychosis: reliability, validity and sensitivity to change. *Acta Psychiatrica Scandinavica* **89**, 62–67

**Boyd Ritsher J, Otilingam PG and Grajales M** (2003) Internalized stigma of mental illness: psychometric properties of a new measure. *Psychiatry Research* **121**, 31–49

**Boyer L, Simeoni M-C, Loundou A, D’Amato T, Reine G, Lancon C and Auquier P** (2010) The development of the S-QoL 18: a shortened quality of life questionnaire for patients with schizophrenia. *Schizophrenia Research* **121**, 241–250

**Breiman L** (2001) Random Forests. *Machine Learning* **45**, 5–32

**Breiman L, Friedman JH, Olshen RA and Stone CJ** (1984) Classification and regression trees. Belmont, CA: Wadsworth. *International Group* **432**, 9

**Chen T and Guestrin C** (2016) Xgboost: A scalable tree boosting system. In *Proceedings of the 22nd acm sigkdd international conference on knowledge discovery and data mining*, pp. 785–794

**Friedman J, Hastie T and Tibshirani R** (2010) Regularization paths for generalized linear models via coordinate descent. *Journal of statistical software* **33**, 1

**Greenwell B** (2021, December 6) fastshap: Fast Approximate Shapley Values

**Guy W** (1976) ECDEU assessment manual for psychopharmacology. US Department of Health, Education, and Welfare, Public Health Service, Alcohol, Drug Abuse, and Mental Health Administration, National Institute of Mental Health, Psychopharmacology Research Branch, Division of Extramural Research Programs

**Kuhn M** (2014) Futility analysis in the cross-validation of machine learning models. *arXiv preprint arXiv:1405.6974*

**Lundberg SM and Lee S-I** (2017) A unified approach to interpreting model predictions. *Advances in neural information processing systems* **30**

**Phillips RV, van der Laan MJ, Lee H and Gruber S** (2022) Practical considerations for specifying a super learner. *arXiv preprint arXiv:2204.06139*

**Rubin DB** (1976) Inference and missing data. *Biometrika* **63**, 581–592

**Stone M** (1974) Cross-Validatory Choice and Assessment of Statistical Predictions. *Journal of the Royal Statistical Society: Series B (Methodological)* **36**, 111–133

**Štrumbelj E and Kononenko I** (2014) Explaining prediction models and individual predictions with feature contributions. *Knowledge and information systems* **41**, 647–665

**Tennant R, Hiller L, Fishwick R, Platt S, Joseph S, Weich S, Parkinson J, Secker J and Stewart-Brown S** (2007) The Warwick-Edinburgh Mental Well-being Scale (WEMWBS): development and UK validation. *Health and Quality of Life Outcomes* **5**, 63
